# Coupling the electrocatalytic dechlorination of 2,4‐D with electroactive microbial anodes

**DOI:** 10.1111/1758-2229.13187

**Published:** 2023-07-21

**Authors:** Luis F. Leon‐Fernandez, Xochitl Dominguez‐Benetton, José Villaseñor Camacho, Francisco Jesús Fernandez‐Morales

**Affiliations:** ^1^ Chemical Engineering Department, ITQUIMA University of Castilla‐La Mancha Ciudad Real Spain; ^2^ Separation and Conversion Technologies Flemish Institute for Technological Research (VITO) Mol Belgium

## Abstract

This work proves the feasibility of dechlorinating 2,4‐D, a customary commercial herbicide, using cathodic electrocatalysis driven by the anodic microbial electrooxidation of sodium acetate. A set of microbial electrochemical systems (MES) were run under two different operating modes, namely microbial fuel cell (MFC) mode, with an external resistance of 120 Ω, or microbial electrolysis cell (MEC) mode, by supplying external voltage (0.6 V) for promoting the (bio)electrochemical reactions taking place. When operating the MES as an MFC, 32% dechlorination was obtained after 72 h of treatment, which was further enhanced by working under MEC mode and achieving a 79% dechlorination. In addition, the biodegradability (expressed as the ratio *BOD/COD*) of the synthetic polluted wastewater was tested prior and after the MES treatment, which was improved from negative values (corresponding to toxic effluents) up to 0.135 in the MFC and 0.453 in the MEC. Our MES approach proves to be a favourable option from the point of view of energy consumption. Running the system under MFC mode allowed to co‐generate energy along the dechlorination process (−0.0120 kWh mol^−1^), even though low removal rates were attained. The energy input under MEC operation was 1.03 kWh mol^−1^—a competitive value compared to previous works reported in the literature for (non‐biological) electrochemical reactors for 2,4‐D electrodechlorination.

## Graphical abstract

This study demonstrates the successful dechlorination of 2,4‐D herbicide using microbial electrochemical systems (MES): Microbial Fuel Cell (MFC) and Microbial Electrolysis Cell (MEC). The MEC achieved higher dechlorination rates (79%) compared to the MFC (32%). The MES approach offers competitive removal rates, lower energy consumption, and reduced capital expenditure compared to abiotic electrochemical technologies, making it a promising alternative. A conceptual scheme is proposed to assess the profitability of MES over conventional electrochemical approaches.

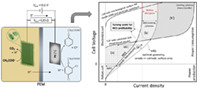

## INTRODUCTION

Nowadays, a broad spectrum of pesticides and herbicides are used in the soil exploitation from agricultural activities. These chemicals are based on organic molecules with antimicrobial properties, standing out among them the organochlorinated compounds (Wen et al., 2013; Ya et al., 2017). In addition, these organics are applied in many other uses, such as synthesizing phenolic resins, paper, and dyes. Organochlorinated compounds feature intense odour, high toxicity, and low biodegradability. The significance of these characteristics increases as a function of the number and position of the chlorines in their aromatic ring (Igbinosa et al., 2013). Due to their extensive and continued use, organochlorinated compounds are almost everywhere. Their persistence in the environment has led to critical events of soil pollution and, consequently, ground and superficial water bodies receiving the water runoff therefrom. As a consequence of the problems related to the environmental emission of organochlorines, the development of removal technologies has received significant attention during the last decade (Aristov & Habekost, 2010). Among other organochlorinated compounds, the polar and slightly volatile herbicide 2,4‐dichlorophenoxyacetic acid (2,4‐D) is globally used in agricultural activities, as it avoids the growth of unwanted grassy weeds in crops, therefore increasing their yield (Cao et al., 2016; Carboneras et al., 2017).

In scientific and technical literature, different processes have been proposed for the removal of organochlorinated pollutants. Among them, microbial processes stand up as landmark technologies due to their low cost and high sustainability. Unfortunately, long‐lasting lag phases and hydraulic retention times are required to remove organochlorines (Field & Sierra‐Alvarez, 2008; Rodriguez et al., 2013). Because of these drawbacks, other removal technologies such as Advanced oxidation processes (AOPs) have been developed. 2,4‐D has been successfully degraded by different techniques such as (photo)(electro)fenton (Cai et al., 2022; Zhu et al., 2020), anodic oxidation (Cai et al., 2021; Fontmorin et al., 2012; Souza et al., 2016) and ozonisation (Bradu et al., 2017). Most of these electrochemical treatments involve the production of highly reactive hydroxyl radicals (^•^OH), which exhibit non‐selective reactivity towards a wide range of organic molecules. Unfortunately, they require costly catalysts and, if the oxidation is not fully efficient, by‐products that are more toxic than the original pollutant could be generated (Forti et al., 2020; Garba et al., 2019; Sun et al., 2014).

Other treatments involve the chemical reduction and dechlorination of the organochlorines, which were also successfully applied to 2,4‐D (Wu et al., 2021; Zhou et al., 2018). In this case, the chlorinated compounds are not mineralized; yet, they generate by‐products presenting lower toxicity and higher biodegradability than the parent compounds, which could be easily oxidized by using other low‐cost technologies such as biological treatments (Descorme, 2017). Recently, electrochemical reductive dechlorination (ERDC), based on the cleavage of the chlorines bonded to the molecule taking place on a cathode, stands up as a promising technology. Moreover, it can be implemented with low investments and mild operating conditions (Zhou et al., 2019).

The ERDC of chlorinated compounds involves a mechanism known as electrocatalytic hydrogenolysis (ECH) (Zhou et al., 2019), described by reactions (1), (2), (3), (4) (Chen et al., 2006; He et al., 2016).

(1) $$2H_{2}O+2e^{-}+M\to 2{\left(H^{•}\right)}_{\mathrm{ads}}M+2{\mathrm{OH}}^{-}$$

(2) $$R–\mathrm{Cl}+M⇄{\left(R–\mathrm{Cl}\right)}_{\mathrm{ads}}M$$

(3) $${\left(R–\mathrm{Cl}\right)}_{\mathrm{ads}}M+2{\left(H^{•}\right)}_{\mathrm{ads}}M\to {\left(R–H\right)}_{\mathrm{ads}}M+\mathrm{HCl}$$

(4) $${\left(R–H\right)}_{\mathrm{ads}}M⇄R–H+M$$

In the ECH process, protons (H^+^) in aqueous solution are reduced to atomic hydrogen (H^•^, a strong reducing agent) on the cathode surface, which subsequently attacks and cleaves C—Cl bonds to achieve hydrodechlorination. H^•^ may also evolve into molecular hydrogen (H_2_) as a side reaction at a more reductive potential, competing with the ECH pathway (Jiang et al., 2017). Operating variables including electrode material, applied potential, and *pH*, are essential for achieving efficient dechlorination yields in this process (Jiang et al., 2018; Peters et al., 2014). Pd, Pt, and Ni are common catalysts for ECH, and among them, Pd is the most relevant one due to its excellent capacity to absorb molecular hydrogen into its lattice structure (Sun et al., 2011). However, the main drawback of ERDC technology is its significant energy consumption and the need to use expensive catalysts, which are critical raw materials for that matter. Few studies are reported in the scientific literature dealing with the 2,4‐D electrocatalytic dechlorination on cathodes based on the aforementioned catalyst materials (Sun et al., 2015; Xu et al., 2013).

In contrast, the development of microbial electrochemical systems (MESs) might reduce the electricity consumption of the ERDC by coupling both technologies, microbial and electrochemical. Once coupled, the MES abates the energy requirements, as the microbial culture enables achieving the ERDC at reduced anode potentials (Wen et al., 2013), and hence dechlorination is expected to be attained at lower cell potentials.

MESs are usually based on a biotic anode and an abiotic cathode. At the biotic anode, the electrogenic culture forms a biofilm carrying out the (bio)electrooxidation of organics, releasing electrons to the solid‐state electrode. At the abiotic cathode, an electrochemical reduction reaction takes place consuming the electrons generated at the anode. Depending on the thermodynamic balance of both half‐reactions, two situations can be identified in the MESs. When both half‐reactions, anodic and cathodic, are spontaneous, the chemical energy is converted into electrical energy. These MESs systems are called Microbial Fuel Cells (MFCs). In contrast, when the reactions are not spontaneous and it is necessary to supply additional energy to operate the system and transform the reactants into products, these MESs are called Microbial Electrolysis Cells (MECs) (Wang & Ren, 2013). Even in the latter case, operating as MEC, the energy contribution is interesting, as the energy consumed by a MECs is much lower than the energy consumed by a conventional electrochemical system (e.g., by 1–5 times), due to the electrical contribution of the electrogenic culture (Wen et al., 2013).

In this context, the present work studies the electrocatalytic hydrodechlorination of 2,4‐D on a Pd‐based cathode driven by anodic biocatalysis in a so‐called MES, operating it under MFC or MEC mode, this latter case to promote the dechlorination rates. Microbial electrochemical dechlorination of organichlorines has been previously introduced (Zhu et al., 2022), both in biological (Fernández‐Verdejo et al., 2021; Lin et al., 2019; Zeppilli et al., 2021; Zhang et al., 2021) and, less intensively, in non‐biological cathodes (Gusseme et al., 2012; Wen et al., 2013), which is the focus of our work; however, to the authors' knowledge, the electrocatalytic hydrodechlorination of 2,4‐D in the abiotic cathode of a MES not been an object of study thus far.

## MATERIALS AND METHODS

### Experimental set‐up

In this work, for the sake of reproducibility, three replicates of the MESs were used. Parallelly, a blank test with an abiotic anode was used to quantify the non‐biological processes taking place in the system. In this way, the biological contribution can be identified by differences between the biotic reactor and the abiotic reference test. The MESs used in this work consisted of two chambers of 0.1 L volume each. These were separated by a proton exchange membrane (Nafion® 117, DuPont). The anode was made of carbon felt (KFA10, SGL Carbon Group®), with a specific area of 3.53 × 10^5^ cm^2^ g^−1^ and a porosity of 0.95 (Asensio et al., 2017). In the case of the anode, this porosity allowed a proper biofilm development, facilitating the biochemical reactions (Leon‐Fernandez et al., 2019). The dimensions of the carbon felt anode were 2.5 cm × 2.5 cm × 0.8 cm. The cathode was carbon cloth (Fuel Cell Store) with dimensions of 2.5 cm × 2.5 cm, onto which palladium was deposited by drop‐casting. As previously described in the introduction, Pd was selected over Pt and Ni materials due to its superior ability to absorb molecular hydrogen into its lattice structure, making it the ideal material for promoting the electrocatalytic dechlorination reactions (Sun et al., 2011). The catalyst ink used for the Pd deposition on the cathode was prepared with commercial 40% Pd/C Vulcan XC‐72R Carbon Black (Fuel Cell Store), Nafion ionomener (alcohol‐based dispersion at 5 wt. %), and isopropanol as a dispersing solvent, following the procedure reported by (Calcerrada et al., 2019). The ratio 40% Pd/C and Nafion solution was 1:8 and the catalyst loading on the cathode was 0.5 mg Pd cm^−2^.

**FIGURE 1:**
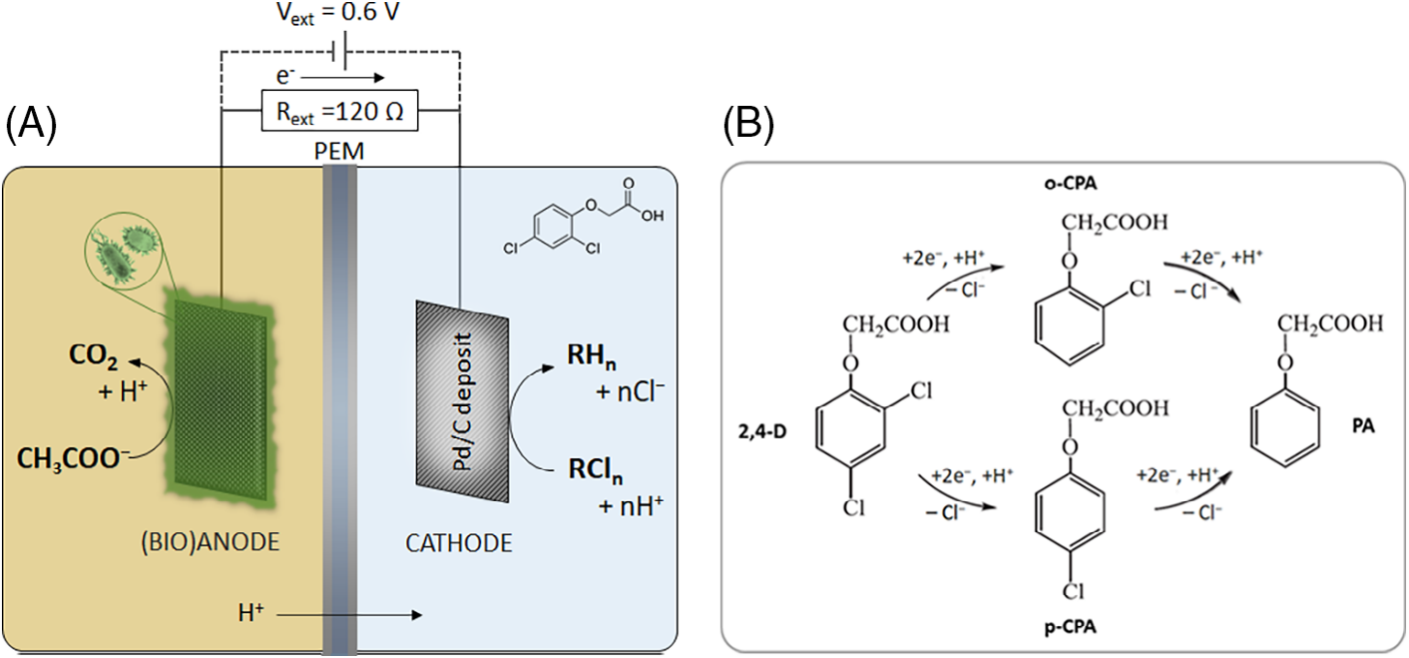
(A) Conceptual description of the MES reactor. (B) Expected by‐products of the dechlorinating process taking place on the cathode through electrocatalytic hydrogenation (ECH) mechanisms.

The MESs studied in this work and their corresponding abiotic reference tests were operated at 25°C. Regarding the operational mode, MFC and MEC configurations were studied in batch operating mode. On the one hand, when running the MESs as MFCs, the anode and the cathode were electrically connected, employing an external load of 120 Ω which guarantees achieving sufficiently negative potentials to accomplish the electrocatalytic dechlorination reactions, as explained in greater detail in the results section. On the other hand, when operating MES as MECs to promote the dechlorination rate, the anode and the cathode were connected to a power supply with a 0.6 V input. A schematic representation of the experimental setup used in this study is presented in Figure 1A. The expected dechlorination pathways and corresponding by‐products are shown in Figure 1B. In the diagram, 2,4‐D represents 2,4‐dichlorophenoxyacetic acid, p‐CPA (or 4‐CPA) represents 4‐chlorophenoxyacetic acid, o‐CPA (or 2‐CPA) represents 2‐chlorophenoxyacetic acid, and PA represents phenoxyacetic acid.

The crystal structure of the Pd/C modified cathode was determined using X‐ray diffraction (XRD). A Philips PW‐1700 X‐ray diffractometer equipped with a rotating anode and utilizing Cu Kα radiation (λ = 1.5413 Å) was employed for the analysis. Figure S1 reports the X‐ray diffraction analysis of the cathode, exhibiting the peaks of Pd^0^.

### Anolyte and catholyte compositions

A 300 ppm 2,4‐D synthetic solution was used as a catholyte. A 100 mmol L^−1^ phosphate solution was employed as a buffer and supporting electrolyte, maintaining the *pH* at 7. It contained 8.66 g L^−1^ of Na_2_HPO_4_ and 5.31 g L^−1^ of KH_2_PO_4_ (Leon‐Fernandez et al., 2021; Wen et al., 2013). The conductivity of the synthetic solution was 11.2 mS cm^−1^. Polluted groundwaters will most likely have ionic conductivities below 1 mS cm^−1^ (Fiori, 2017), lower than the simulated 2,4‐D polluted streams we addressed in this work, which might lead to a high internal resistance in the MES (high IR drop in the electrolyte). However, this could be easily circumvented by adding a supporting electrolyte (e.g., NaCl), or by improving the architecture of the reactor (minimizing the distance between anode and cathode). As the dechlorination reactions and ECH mechanisms are *pH* dependent, for the sake of experimental reproducibility, we use the phosphate buffer composition described above in order to avoid *pH* changes in the catholyte, which also provided sufficient ionic conductivity.

The microbial growth medium used as an anolyte consisted of CH_3_COONa 1 g L^−1^, Na_2_HPO_4_ 3 g L^−1^, KH_2_PO_4_ 0.7 g L^−1^, (NH_4_)_2_SO_4_ 0.8 g L^−1^, MgSO_4_·7H_2_O 0.2 g L^−1^, and (NH_4_)_2_Fe(SO_4_)_2_·6H_2_O 0.04 g L^−1^ (Izadi et al., 2019; Leon‐Fernandez et al., 2021). The *pH* and conductivity of the fresh anolyte were 7.44 and 5.7 mS cm^−1^, respectively.

In the abiotic cells (blank experiments), 100 ppm of sodium azide were added to the anolyte formulation, to avoid bacterial growth (Cabrol et al., 2017).

### Start‐up and operation of the MESs

The anodic compartment of the MESs were inoculated with a microbial consortium from a previously running acetate‐fed MFC, whose initial seed was obtained from the aerobic reactor of a conventional activated sludge facility (volatile suspended solids concentration of approximately 2300 mg L^−1^, hydraulic retention time of 5 h and sludge retention time of 7 days (Mateo et al., 2018)). The methodology for biofilm development was previously described elsewhere (Leon‐Fernandez et al., 2021). Briefly, the anolyte in the experimental setup was initially filled with the effluent from a previously operated microbial fuel cell (MFC). Subsequently, 80% of the anolyte was replaced with fresh medium every 2 days. The MESs was run as MFCs, using a 120 Ω external resistor. The cathodic reaction for such a start‐up operation was the oxygen reduction reaction, ORR (by bubbling O_2_ in the catholyte, using the catholyte composition described above but without 2,4‐D). After one week, the electrical power output was stable, reaching a maximum cell voltage of 0.24 V (around 2 mA). The OCV value was 0.55 V and the anode OCP value was −0.495 V_Ag/AgCl_.

After the MESs start‐up, the bioanodic electrooxidation of sodium acetate was coupled with the cathodic electrocatalytic dechlorination of 2,4‐D. To prevent the presence of oxygen in both the anolyte and catholyte, N_2_ was sparged for 10 min in both compartments prior to the batch experiments.

### Analytical measurements

High‐performance liquid chromatography (HPLC) (Jasco, Japan) operated with a column Kinetex 5 μm Biphenyl 100 Å, 150 mm × 4.5 mm (Phenomenex, Torrance, CA, USA) was used to detect and quantify the chlorinated compounds.

HPLC analysis was also used for sodium acetate quantification; in this case, an Agilent 1260 Infinity equipment containing a Hi‐plex H column (300 mm × 7.7 mm, 8 μm) was employed. The mobile phase consisted of 5 mmol L^−1^ H_2_SO_4_, the wavelength was 210 nm, and the flow rate 0.4 mL min^−1^.

A compact ion chromatograph coupled to a conductivity detector (Metrohm 930) was used to quantify the chloride concentration. A Metrosep A Supp 7 column and a 15:85 v/v acetone/3.6 mM Na_2_CO_3_ mobile phase at a flow rate of 0.8 cm^3^ min^−1^ were employed.

The mass balance resolved for the organochlorines and the chloride generation was verified along the experiments, as shown in Equations (5) and (6).

(5) $$\begin{array}{l} \sum {\text{[Organochl. ]}}_{t}\\ \;={\left[2,4–D\right]}_{t}+{\left[4–\mathrm{CPA}\right]}_{t}+{\left[2–\mathrm{CPA}\right]}_{t}+{\left[\mathrm{PA}\right]}_{t}\\ \;=\sum {\left[2,4–D\right]}_{t_{0}} \end{array}$$

(6) $${\left[{\mathrm{Cl}}^{-}\right]}_{t}={\left[4–\mathrm{CP}\right]}_{t}+{\left[2–\mathrm{CP}\right]}_{t}+2\times {\left[\text{phenol}\right]}_{t}$$

where ${\left[2,4-D\right]}_{t}$, ${\left[4-\mathrm{CPA}\right]}_{t}$, ${\left[2-\mathrm{CPA}\right]}_{t}$, ${\left[\mathrm{PA}\right]}_{t}$ and ${\left[Cl^{-}\right]}_{t}$ are the concentrations of 2,4‐dichlorophenoxyacetic acid, 4‐chlorophenoxyacetic acid, 2‐chlorophenoxyacetic acid, phenoxyacetic acid and chloride at a certain time t.

The *dechlorination yield (%)* was calculated according to Equation (7) (concentration in mol L^−1^).

(7) $$\text{Dechlor.yield}\left(\%\right)=\frac{{\left[{\mathrm{Cl}}^{-}\right]}_{t}}{2\times {\left[2,4–D\right]}_{t_{0}}}\times 100$$

Pseudo‐first‐order kinetics to assess the 2,4‐D removal/dechlorination rate were proposed. The pseudo‐first‐order kinetic constant was obtained by fitting the experimental data with Equation (8), which corresponds to the integrated mass balance discontinuous reactor and for a first‐order kinetics reaction:

(8) $$\left[2,4–D\right]={\left[2,4–D\right]}_{0}\times e^{-k_{\mathrm{obs}}\times t}$$

where $\left[2,4–D\right]$ is the 2,4‐D concentration in mmol L^−1^ at time t (min), ${\left[2,4-D\right]}_{0}$ is the 2,4‐D initial concentration in mmol L^−1^ and $k_{\mathrm{obs}}$ is the pseudo‐first‐order kinetic constant for the reaction in min^−1^ (the fitted parameter).

Conductivity and *pH* were determined by using a Crison Cm 35 and a GLP22 Crison sensors, respectively.

Chemical oxygen demand (*COD*) was measured using a colorimetric method employing Spectroquant® *COD* Test Kits. The samples underwent heating at 148°C for 120 min using a Velp ECO‐16 thermoreactor, and the absorbance measurements were conducted using a Spectroquant® Pharo 100 Merck spectrophotometer.

The Biochemical Oxygen Demand (*BOD*) was determined by using an Oxitop IS 6‐Var. Activated sludge from a conventional wastewater treatment plant was used as inoculum for the *BOD* tests (Leon‐Fernandez et al., 2021). In order to inhibit bacterial nitrification metabolic routes, two drops of N‐Allylthiourea were added to the samples.

The Biochemical Oxygen Demand (*BOD*) at t=∞ was calculated according to Equation (9), where *Y* is the *BOD* at a certain time t, *t* is the time in days, *BOD*_*f*_ is the *BOD* reached at t=∞ in mg L^−1^ and k is the biodegradation constant in d^−1^. The constant k gives an idea of the kinetics of the biodegradation test. *BOD*_*f*_ and *k* parameters were estimated through a fitting of the experimental data obtained in the *BOD*_*5*_ tests performed to the wastewater at the beginning and at the end of the (bio)electrochemical dechlorination treatment.

(9) $$Y={\mathrm{BOD}}_{f}\times \left(1-e^{-\mathrm{kt}}\right)$$

The inhibitory effect of the sample on the light emission of luminescent bacteria, *Aliivibrio fischeri*, is measured with a luminometer (Junior LB 9509 of Berthold Technologies). The toxicity was evaluated as *EC*_*50*_ by measuring the effective concentration that produces a 50% inhibition calculated from the loss of luminescence (Rodriguez et al., 2013).

### Electrochemical analyses

Linear sweep voltammetries (LSV) were performed at 25°C, at a scan rate of 0.5 mV s^−1^, and starting at open circuit potential (OCP), using a potentiostat/galvanostat (Autolab, PGSTAT‐302 N). LSVs were not conducted in the MES reactor but in a half‐cell with constant magnetic stirring of 350 rpm when using the carbon cloth electrode with 0.5 mg cm^−2^ Pd loading (cathode of the MES) as the working electrode (WE), and 60 rpm in the case of using the carbon felt electrode with electroactive biofilm (anode of the MES) as the WE. An Ag/AgCl (in 3 M KCl) electrode and a Pt wire were used as reference (RE) and counter electrode, respectively.

Polarization and power curves of the MFC were obtained by modifying the external resistance. The set of resistances employed is next: 100 GΩ, 38.78 kΩ, 26.75 kΩ, 11.82 kΩ, 5.53 kΩ, 3.30 kΩ, 2.18 kΩ, 1.19 kΩ, 814 Ω, 553 Ω, 464 Ω, 267 Ω, 118.3 Ω, 56.5 Ω, 22 Ω, 14.7 Ω, 6.9 Ω.

During the bio‐electro dechlorination experiments, electrical current was recorded with a Keithley 2000 multimeter. The resulting current densities were calculated with respect to the projected cathodic area, given that this is the limiting electrode in the systems studied.

The cathode and anode potential of the MESs were measured against an Ag/AgCl RE. Before the experiments, nitrogen gas was sparged into the solutions in order to desorb the dissolved oxygen.

The current efficiency for the dechlorination reactions (*CE*, Equation 10) and faradaic efficiency for acetate oxidation (*FE*, Equation 11) for the given time interval were calculated as follows (Pletcher & Walsh, 1990).

(10) $$\mathrm{CE}\left(\%\right)={\left(\frac{\int_{t_{1}}^{t_{2}}Idt}{\mathrm{F\sum}\left(n_{i}∆m_{i}\right)}\right)}^{-1}\times 100$$

(11) $$\mathrm{FE}\left(\%\right)=\left(\frac{\int_{t_{1}}^{t_{2}}Idt}{\mathrm{F\sum}\left(n_{i}∆m_{i}\right)}\right)\times 100$$

wherein *I* is the electric current in A, *t* is the time in seconds, F is the Faraday's constant (96485.3 C mol^−1^), n_i_ is the number of electrons involved in the corresponding electrochemical reaction per mol of reactant, and *Δm*_*i*_ is the molar variation of reactant *i* during the time interval of integration (2,4‐D, 4‐CPA and 2‐CPA for the case of *CE*, and sodium acetate for the case of *FE*) in mol.

The energy and power consumption were calculated according to Equations (12) and (13), respectively:

(12) $$E=\frac{\left(\int_{0}^{t}Pdt\right)}{∆m_{2,4–D}}$$

(13) $$P=V_{\text{cell}}\times I$$

where E is the energy consumed kWh mol^−1^, $∆m_{2,4–D}$ is the 2,4‐D removal in mol, P is the power in W (kW by dividing by 1000), $V_{\text{cell}}$ is the cell voltage in V, and I is the current in A.

## RESULTS AND DISCUSSION

The cathodic dechlorination process is closely linked to the electrocatalytic hydrogenation (ECH) mechanism, which requires H^+^ reduction on the cathode. Hence, cathodic potential values that allow H^+^ reduction will be required to achieve the 2,4‐D dechlorination in the MES. Our previous works described that it is possible to work under MFC mode, as the starting potential for the ECH reactions at the cathode (*E*_*cath*_) is over the starting potential for the microbial oxidation of acetate ions at the anode (*E*_*an*_), that is, *E*_*cath*_ > *E*_*an*_ (Leon‐Fernandez et al., 2020; Leon‐Fernandez et al., 2021). Figure S2a and S2b show the corresponding anodic and cathodic operative curves. The anodic one (LSV performed on the carbon felt electrode with electroactive bacteria developed on it at its growth phase steady‐state performance) corresponds to the (bio)oxidation of sodium acetate, 1 g L^−1^. On the other hand, in the cathodic operative curve, different reactions occur. First, a plateau region is reached within the voltage range of 0.25 to −0.17 V_Ag/AgCl_, which is likely associated with the cathodic deprotonation of phosphates present in the solution and mass transport limitation of protons to the electrode at neutral *pH* values (Da Silva et al., 2004). From potentials more negative than −0.17 V_Ag/AgCl_, H_2_ evolution (HER) and the ECH mechanism occur, resulting in the dechlorination reactions. Figure S2c shows the polarization and power curves performed on the MFC, whereas Figure S2d shows the anode and cathode potentials throughout this test. The use of a 120 Ω eternal resistor allowed to reach sufficiently negative cathode potentials to promote ECH reactions (i.e., −0.4 V_Ag/AgCl_, more negative than the threshold of −0.17 V_Ag/AgCl_ required for ECH to occur), also maximizing the power output according to the power curve presented in Figure S2c. If the dechlorination reaction rate was to be promoted, an external voltage input (microbial electrolysis mode, MEC) would be required. Working under MEC mode would allow achieving more negative cathode potentials and higher current densities, boosting the ECH reactions. However, as displayed in Figure S2b, the operative (polarization) curve for the (bio)anode reaches limiting current conditions at potentials more positive than −0.2 V_Ag/AgCl_ (i.e., ~0.7 mA cm^−2^). When a voltage input of 0.6 V was applied to the MES, the anode potential rose up to values around −0.2 V_Ag/AgCl_. Therefore, a cell voltage beyond 0.6 V in the MEC would not lead to significantly higher current densities nor faster removal rates due to limitations on the bioanode performance. In addition, very positive anode potentials (or large current densities) might lead to the electroactive biofilm disruption and death, attributed to the evolution of local acidic *pH*, high concentration of oxidants, and the generation of gases at the electrode (such as O_2_ from water splitting or Cl_2_ from Cl^─^ oxidation, which disproportionates in water and gives ClO^─^; Hoseinzadeh et al., 2020). Because of the aforementioned reasons, a 120 Ω external resistor was selected for MFC operation, and an external voltage input of 0.6 V was applied in MEC mode.

**FIGURE 2:**
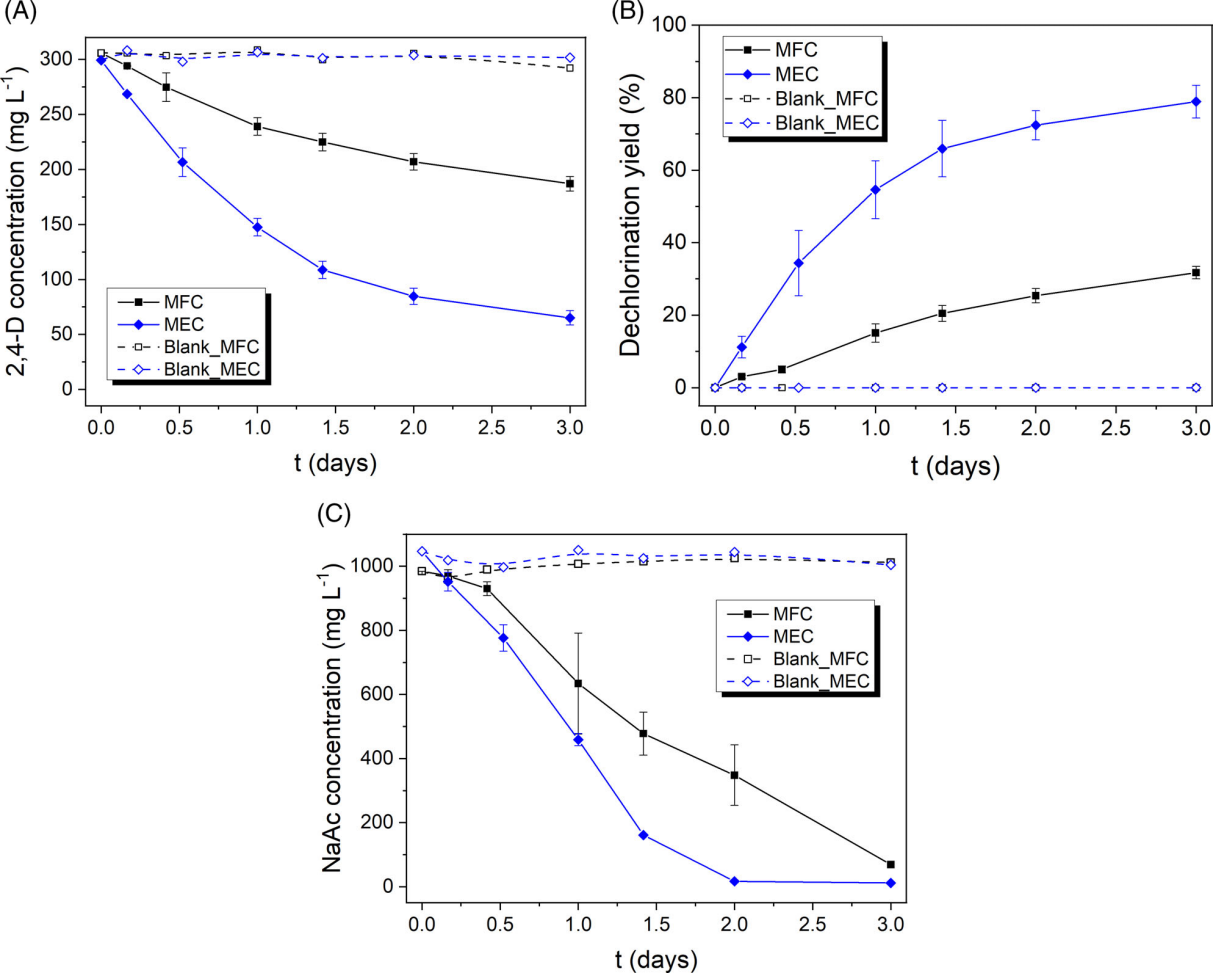
(A) 2,4‐D evolution over time; (B) dechlorination yield over time; (C) sodium acetate evolution over time, for all the systems investigated: Microbial Fuel Cell (MFC), Microbial Electrolysis Cell (MEC), and blank systems with abiotic anode.

### Evaluation of the cathodic dechlorination in the MESs

The expected overall dechlorinating reactions taking place on the Pd‐modified carbon cloth electrode through the ECH mechanism (described in the introduction section, reactions (1), (2), (3), (4)), are presented in reactions 14 and 15. In these equations, x‐CPA stands for the isomers 2‐chlorophenoxyacetic acid (2‐CPA) and 4‐chlorophenoxyacetic acid (4‐CPA).

(14) $$2,4–D+2H^{+}+2e^{-}⇄x–\mathrm{CPA}+{\mathrm{Cl}}^{-}$$

(15) $$x–\mathrm{CPA}+2H^{+}+2e^{-}⇄\mathrm{PA}+{\mathrm{Cl}}^{-}$$

As the starting potential to accomplish the ECH mechanism at pH=7 (i.e., −0.17 V_Ag/AgCl_, refer to Figure S2a) is more positive than the starting potential for the oxidation of sodium acetate under the conditions previously described in Section 2.2 (i.e., −0.495 V_Ag/AgCl_, see also Figure S2b), the cathodic electrocatalytic dechlorination of 2,4‐D coupled with bio‐catalytic oxidation of sodium acetate is feasible under MFC mode, and could be further enhanced by working under MEC mode, polarizing the cathode at more reductive potentials (Leon‐Fernandez et al., 2021).

Hence, two batch experiments were run (in triplicate), operating in first instance under MFC mode with an external resistance of 120 Ω and, later, under MEC mode with an external voltage input of 0.6 V. Additionally, an abiotic reference test (blank) was run in both cases to isolate the effect of the electroactive microorganisms at the anode.

Figure 2 shows the 2,4‐D evolution in the MESs (a), the dechlorination yield (%) (b), and the acetate concentration profiles throughout the batch processes (c).

**FIGURE 3:**
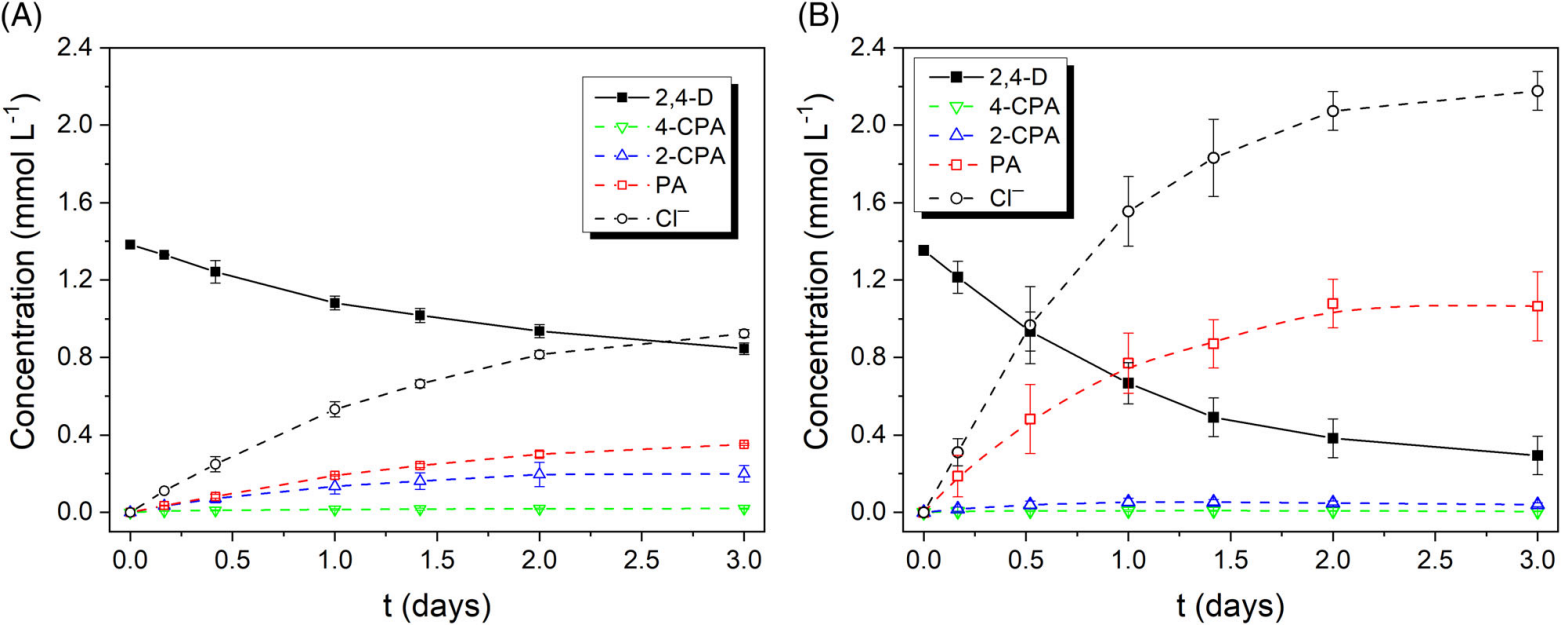
Evolution of organochlorinated species and chlorides generated throughout the dechlorination batch experiments. (A) MFC mode ($r_{\mathrm{ext}}=120\;\Omega$); (B) MEC mode ($V_{\text{cell}}=0.6\;V)$.

During the experiments, the mass balance for organochlorines and chloride generation was carefully monitored and validated using Equations (5) and (6). Any minor discrepancies in the mass balance, typically below 3%, were attributed to analytical errors and slight volatilization of the organochlorines that occurred after sampling and during the course of the experiment. Even though 2,4‐D crossover from catholyte to anolyte and volume variation in the anolyte and catholyte compartments due to osmosis were considered for the dechlorination yield calculation, these effects were not significant throughout the experiments (contribution lower than 0.5% to the overall results).

By supplying external voltage, the removal rate for 2,4‐D was further promoted (see Figure 2A 2,4‐D evolution over time) accompanied by the generation of phenoxyacetic acid, as presented in Figure 3 (concentration of the different organochlorine products over time). Moreover, the voltage input under MEC mode stimulated the anodic biooxidation of sodium acetate through extracellular electron transfer pathways, leading to its depletion after 2 days of operation (Figure 2C) and higher faradaic efficiencies, as will be detailed later in Section 3.3. On the other hand, in the control tests with a non‐biological anode, the concentrations of 2,4‐D and sodium acetate remained nearly unchanged. This confirms that the bioelectrocatalysis at the anode is responsible for the oxidation of sodium acetate (the electron donor) and the driving force behind the cathodic reactions.

**FIGURE 4:**
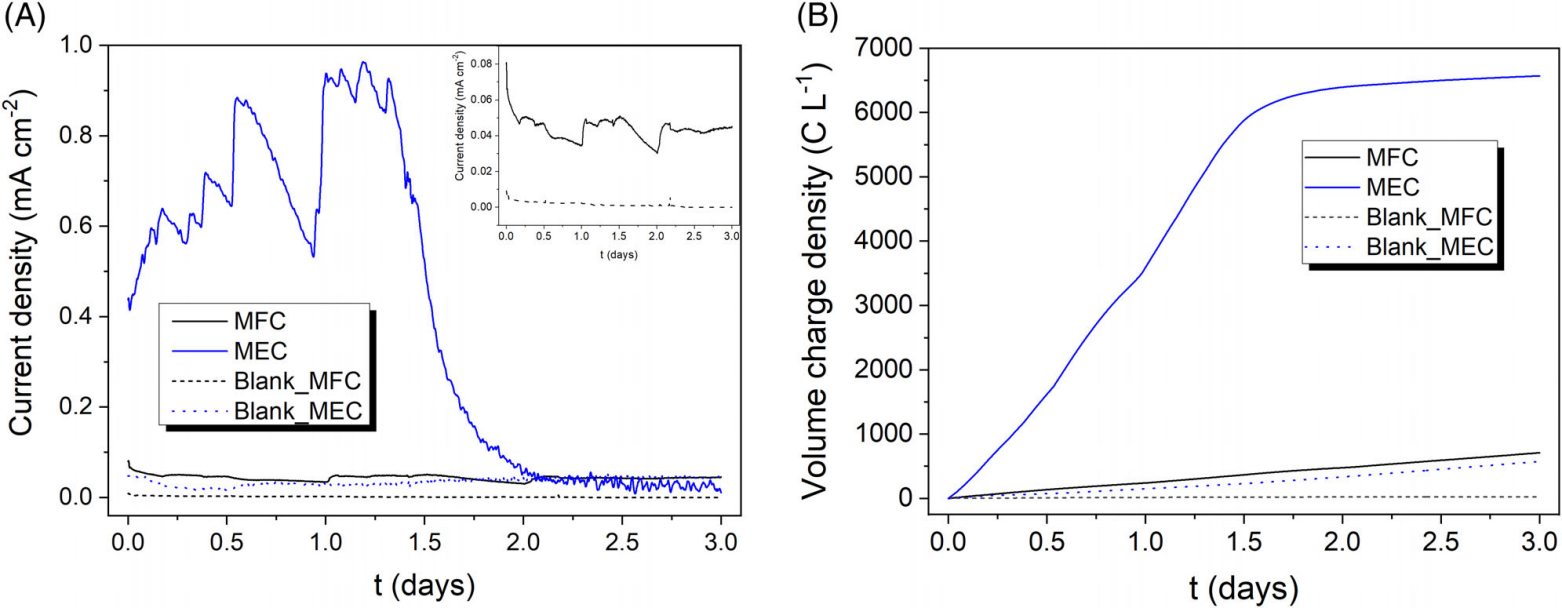
(A) Current density produced by the MESs (average data of the replicates) in the batch experiments under MFC and MEC operating conditions. (B) Volume charge density accumulated over time. The blank experiments correspond to the abiotic reference tests.

**FIGURE 5:**
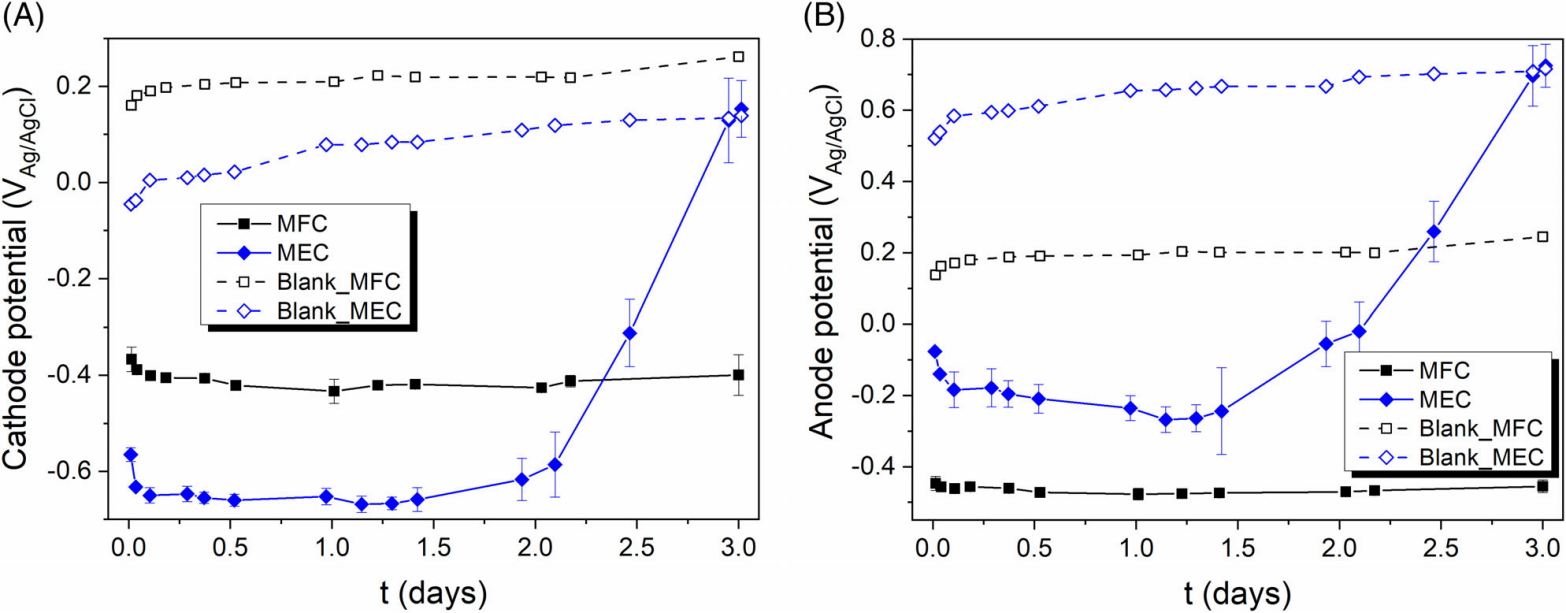
Evolution of (A) cathode potential and (B) anode potential in the MESs. The blank experiments correspond to the abiotic reference tests.

Seventy‐nine percent of 2,4‐D dechlorination was achieved after 3 days under MEC mode, which was notably higher compared to the MFC system, where only 32% dechlorination was achieved (Figure 2B). Before reaching the final dechlorinated product (PA), mono‐chlorinated intermediates were formed (Figure 3). The selectivity towards 2‐CPA was higher than for 4‐CPA. This is explained by the hindering effect (steric limitations) of the $\left[-O{\mathrm{CH}}_{2}\text{COOH}\right]$ chain on the aromatic ring, making the chlorine bound in para‐position more likely to abandon the aromatic ring than the one in ortho‐position, as previously reported in the literature (Sun et al., 2014; Tsyganok & Otsuka, 1999). Furthermore, the accumulation of 4‐CPA and 2‐CPA was less significant in the MEC, implying that by supplying an external voltage input, the dechlorination rate of the mono‐chlorinated intermediates was also promoted (Figure 3B). The generation of phenol and chlorides occurred faster in the MEC, and the amount of Cl^−^ agrees with the mass balance (1 mol of Cl^−^ generated per mol of 2,4‐D dechlorinated to x‐CPA, and 1 mol of Cl^−^ generated per mol of x‐CPA dechlorinated to PA as presented in Equations (6), (14) and (15)).

Similar findings were observed in our previous study (Leon‐Fernandez et al., 2021) where we investigated the dechlorination of 2,4‐dichlorophenol (2,4‐DCP) using the same experimental setup. Table 1 provides the pseudo‐first‐order kinetic constants (*k*_*obs*_) for the removal of both 2,4‐D and 2,4‐DCP under both MFC and MEC modes. The process was favoured in all cases by operating under MEC mode, obtaining higher *k*_*obs*_ values. Nevertheless, It is noteworthy that the 2,4‐D dechlorination rates are significantly lower in comparison with 2,4‐DCP (lower values of the pseudo‐first‐order kinetic constants). The higher steric hindrance of the $\left[-O{\mathrm{CH}}_{2}\text{COOH}\right]$ in the 2,4‐D molecule compared to the hydroxyl group in 2,4‐DCP, and the different formal charge (deprotonation) at a given *pH* likely have a significant impact on the adsorption of the molecule on the Pd active sites (refer to ECH mechanism, reactions (1), (2), (3), (4)). In fact, the *pK*_*a*_ for the deprotonation of the 2,4‐D carboxylic group is 2.73 (Qurratu & Reehan, 2016), whereas for the deprotonation of the 2,4‐DCP hydroxyl group is 7.89 (Muller & Caillard, 2011). Hence, under the given operating *pH* of 7, the [−OH] group of 2,4‐DCP will be mostly protonated, with a neutral formal charge of the molecule ($pK_{a}^{2,4–\mathrm{DCP}}$ > *pH*). On the contrary, the 2,4‐D carboxylic group will be deprotonated ($pK_{a}^{2,4–\mathrm{DCP}}$ < *pH*), having the molecule negative formal charge, which could limit the migration/adsorption onto the cathode (Fontmorin et al., 2012; Tsyganok & Otsuka, 1999). This can explain the more favoured dechlorination kinetics of 2,4‐DCP compared to 2,4‐D. In addition, higher limitations on the 2,4‐D transport from the bulk to the electrode surface compared to 2,4‐DCP might also lead to less favoured dechlorination kinetics (2,4‐D molecule size bigger than 2,4‐DCP). The diffusion coefficient of 2,4‐D and 2,4‐DCP are $0.58\times 10^{-9}\;m\;s^{-1}$ (T=23°C) and $0.78\times 10^{-9}\;m\;s^{-1}$ (T=25°C), respectively (see Table S1 in the supplementary material, where the diffusion coefficient of relevant (chlorinated) aromatic compounds is presented; Martins et al., 2015; Scott & Phillips, 1973).

**TABLE 1:** Comparison of the MES performance of the current work with pure electrochemical approaches investigated in the literature, operating under similar conditions (i.e., electrode surface area, current densities, Pd loading).

Compound	System	Operating conditions	Cathode	Anode	Pseudo first ord. kinetics		Energy consumption/kWh mol^−1^	Average current efficiency/%	Reference
					*k*_*obs*_/min^−1^	r^2^			
2,4‐DCP *C*_*0*_ = 1.83 mmol L^−1^	MFC	*R*_*ext*_ = 120 Ω *j* = 0.13 mA cm^−2^ (max)	Pd/C on carbon cloth *S* = 6.25 cm^−2^ ^a^ Pd content: 0.5 mg cm^−2^ *V*_*cath*_ = 0.1 L	Bioanode (carbon felt)	$7.15\times 10^{-4}$	0.9985	−0.0098 (energy production)	51.12	Leon‐Fernandez et al. (2021)
	MEC	*V*_*cell*_ = 0.6 V *j* = 0.75 mA cm^−2^ (max)			$1.47\times 10^{-3}$	0.9982	0.534	8.44	
2,4‐D *C*_*0*_ = 1.36 mmol L^−1^	MFC	*R*_*ext*_ = 120 Ω *j* ≈ 0.05 mA cm^−2^	Pd/C on carbon cloth *S* = 6.25 cm^−2^ Pd content: 0.5 mg cm^−2^ *V*_*cath*_ = 0.1 L	Bioanode (carbon felt)	$1.33\times 10^{-4}$	0.9703	−0.0120 (energy production)	24.19	Current work
	MEC	(*V*_*cell*_ = 0.6 V) *j* = 0.9 mA cm^−2^ (max)			$4.93\times 10^{-4}$ ^b^	0.9998	1.03	6.83	
2,4‐D *C*_*0*_ = 0.226 mmol L^−1^	Electrolysis (abiotic)	*j* = 0.5 mA cm^−2^	Pd/Ni foam (Pd pulse electrodeposition) Pd loading: 0.67 mg *S* = 4 cm^−2^ ^a^ *V*_*cath*_ = 0.072 L	Platinum foil	$3.17\times 10^{-3}$	0.9495	1.13	10.78	He et al. (2016)
		*j* = 0.5 mA cm^−2^	Pd/Ni foam (electrodeless Pd deposition) Pd loading: 0.68 mg *S* = 4 cm^−2^ ^a^ *V*_*cath*_ = 0.072 L		$7.37\times 10^{-4}$	0.9620	4.99	3.37	
2,4‐D *C*_*0*_ = 0.226 mmol L^−1^	Electrolysis (abiotic)	*j* = 0.83 mA cm^−2^	Nanosized titanium nitride doped palladium/nickel Pd content: 0.44 mg cm^−2^ *S* = 6 cm^−2^ ^a^ *V*_*cath*_ = 0.1 L	Platinum foil	$5.48\times 10^{-3}$	0.9542	2.98	5.80	Sun et al. (2015)

*Note*: Values of the pseudo‐first‐order kinetic constants (*k*_*obs*_, obtained by fitting the experimental data to Equation (8)), energy consumption, and average current efficiency of the different technologies.

^a^ Projected surface area.

^b^ The fitting was carried out with 2,4‐D concentration data until t=1.4d, when there was no substrate limitation at the anode (sodium acetate is depleted in the MEC after t=1.5d, see Figures 2C and 3B).

Figure 4 displays the current density exerted by the MES systems under the different operating modes (a), and the volume charge density used along the process (b). A far higher current density was achieved in the MEC systems (maximum values of about 0.85 mA cm^−2^) compared to MFC mode (maximum values of 0.08 mA cm^−2^, one order of magnitude lower). The fluctuations in the current density experienced during the first 30 h are due to the manual control of the cathode and anode *pH* to 7 and 7.5, respectively. A more frequent *pH* adjustment was required under MEC mode (see Figure 4). In addition, the current under MEC configuration exhibited a gradual decrease after t=1.5days, attributed to the sodium acetate depletion in the anolyte (as shown in Figure 2C). In fact, the dechlorination process experiences a slowdown as the current falls. The dechlorination yield could have been enhanced by ensuring an ample supply of substrate throughout the process, thereby preventing it from becoming a limiting factor. By maintaining an adequate substrate concentration, the dechlorination reactions could have been sustained at a higher rate, resulting in improved performance. The current density exerted by the blank cells (MFC and MEC blanks) was negligible compared to their respective biotic reactors because of the absence of biocatalysis at the anode.

**FIGURE 6:**
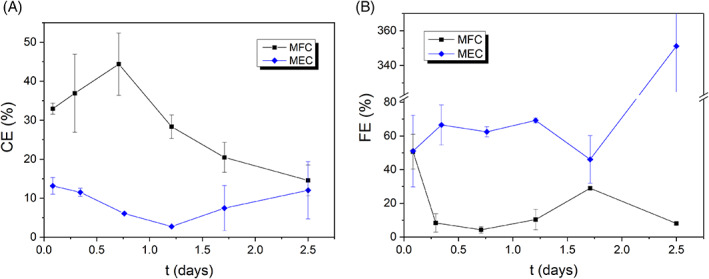
Evolution of the faradic efficiencies in the MESs. (A) Current efficiencies (*CE*), (B) Faradaic efficiencies (*FE*). The data series correspond to the *CE* or *FE* average values for each interval between sample takings (located at the midpoint of their respective interval).

Even though the MEC exhibited a higher current output and, consequently, faster dechlorination rates, the volume charge density (VCD) or charge invested per unit volume is considerably higher in the MEC system compared to the MFC, as depicted in Figure 4B. This translates into a higher power consumption per unit volume (or per mol of 2,4‐D) and a lower current efficiency, as further developed later in Section 3.3 (process efficiency and benchmarking).

Figure 5A,B report on the cathode and anode potential of the MFC and MEC systems, surpassing in both cases the starting potential required to achieve the ECH reactions, that is, more negative values than −0.17 V_Ag/AgCl_. The stable anodic biocatalysis in the MFC allowed for a cathode polarization of approximately −0.4 V_Ag/AgCl_. In contrast, applying an external voltage input under MEC mode facilitated a more negative cathode polarization, reaching approximately −0.65 VAg/AgCl during the initial 2 days and subsequently increasing to 0.15 V_Ag/AgCl_, which led to the cessation of ECH reactions. This change in the cathode potential (and decrease in current, Figure 4A) can be attributed to the faster consumption and depletion of sodium acetate as the electron donor at the anode at t=1.5−2d, as previously mentioned. By polarizing the cathode potential to more negative values under MEC configuration, the ECH reactions are promoted, involving water electrolysis and H^•^ chemisorption on Pd active sites. This leads to faster dechlorination rates, but also an increased hydrogen production (see Figure S2a). As a result, MEC operation leads to higher current densities (Figure 4A), but it is associated with lower faradaic efficiencies, which will be further discussed in Section 3.3.

The cathode potential was always positive in the reference tests with abiotic anode (blank experiments); over 0 V_Ag/AgCl_ in the MEC reference test and close to the equilibrium potential (starting potential of the LSVs, Figure S2a) in the MFC reference tests, establishing around 0.2 V_Ag/AgCl_. Hence, no dechlorination reaction occurred in both MFC and MEC abiotic reference tests.

The anode potential under MEC mode stabilized at more positive values than those for MFC mode, at around −0.2 V_Ag/AgCl_ during the initial 1.5 days. As previously described, the anodic (bio)driven sodium acetate oxidation exhibits limiting current conditions when the anode potential exceeds −0.2 V_Ag/AgCl_, as observed in the LSV depicted in Figure S2b. Therefore, a higher anode polarization or, in other words, a cell voltage beyond 0.6 V in the MEC would not lead to significantly higher current densities nor faster removal rates due to limitations on the bioanode performance (reason why a cell voltage of 0.6 V was deemed optimal for the given set‐up and operating conditions). The anode potential shifted towards more positive values in the MEC after t=1.5days due to the sodium acetate abatement in the anolyte (Figure 2C). In relation to the MFC configuration, the anode potential stabilized at −0.45 V_Ag/AgCl_, indicating milder conditions for the (bio)anode as compared to the MEC. Interestingly, even though the concentration of sodium acetate decreased over time in both the MEC and MFC configurations, the anode potential remained stable in both systems (at all times in the MFC, and during the first 1.5 days in the MEC, when sodium acetate abatement occurred). This stability in the anode potential can be attributed to the excess of sodium acetate, which indicates that it was not a limiting substrate in bioprocess. According to the biological Monod kinetic model, as the substrate concentration increases, there reaches a point where the specific biomass growth or substrate consumption rate plateaus (it is important to note that excessively high substrate concentrations could potentially lead to substrate inhibition, where the growth or consumption rate may be hindered) (Leon‐Fernandez et al., 2022). This explains the stable conditions at the anode as long as the substrate is present in excess and the rest of the operating conditions remain unchanged (e.g., *pH*, counter reaction, cell voltage).

It is noteworthy that in both MES configurations, the anode potential remained sufficiently negative to power/drive the cathode reactions through bioelectrocatalysis. This stands in contrast to the respective blank counterparts in the MFC and MEC systems, where the anode potential was above 0.2 V_Ag/AgCl_ in the MFC blank and 0.6 V_Ag/AgCl_ in the MEC blank, with negligible current generation. It can be concluded that the anodic biocatalysis plays a crucial role in powering the cathodic process in the MFC (achieving spontaneity) or abating the energy requirements in the MEC under the conditions studied in this work.

### Process efficiency and benchmarking

Figure 6A,B report on the current (*CE*) and faradaic (*FE*) efficiencies of the MESs, respectively, for the different operational configurations as per described in Equations (10) and (11). *CE* and *FE* were calculated for each interval between sample takings, and each value was located at the midpoint of its respective interval.

As displayed in Figure 6A, the overall *CE* trend under MFC mode decreased over time due to the decrease in the organochlorine content in the catholyte (i.e., 2,4‐D conversion to fully dechlorinated PA via intermediates 2‐CPA and 4‐CPA), which act as the final electron acceptors. Consequently, the extent of the dechlorination reactions decreased relative to the H_2_ evolution, resulting in lower current efficiencies. The initial lower *CE* values may be attributed to the consumption of trace amounts of dissolved oxygen in the catholyte. On the other hand, when the systems were operated in MEC mode, the cathode potential was polarized towards more negative values. This resulted in higher dechlorination rates but also favoured the H_2_ evolution as a side reaction to a greater, leading to lower cathodic faradaic efficiencies compared to MFC mode. As previously mentioned, when sodium acetate depletion occurred at the anode at t=1.5d, the current started to decrease (as shown in Figure 4A). The increase in *CE* after t=1.5d, coinciding with the decrease in current in the MEC, can be attributed to a higher predominance of ECH mechanism compared to HER at lower current density. This observation is consistent with the higher *CE* achieved in the MFC, which operates at milder conditions.

Regarding the anode performance (Figure 6B), the faradaic efficiencies are significantly below 100% (approximately 20% for MFC mode and around 60% for MEC mode). This can be attributed to the metabolisation of sodium acetate through non‐bioelectrochemical pathways. Some non‐electrochemical pathways may be the partial oxidation of the substrate not leading to CO_2_ (e.g., CH_4_ production), or complete oxidation (aerobic) by traces of O_2_ dissolved in the anolyte. The latter effect was minimized by sparging N_2_ into the anolyte prior to the experiments. The values are in agreement with the literature for bioanodes inoculated with mixed cultures (Beegle & Borole, 2018). The bioanode performance and sodium acetate consumption through extracellular electron transfer pathways (electrogenic pathways) were enhanced by applying an external voltage under MEC mode. This resulted in higher faradaic efficiencies in comparison with the MFC configuration. It is noteworthy that after the second day, the anodic efficiencies in the MEC exhibited a significant increase, surpassing values of 100%. As the sodium acetate concentration was almost depleted during the last 24 h and the current output was also negligible, results from Equation (11) lead to little physical sense values. Nevertheless, as the sodium acetate concentration was almost nil, the electrons generated at the anode are mainly due to endogenous respiration of the electroactive bacteria (stimulated by the external voltage supply), explaining faradaic efficiencies over 100%. Endogenous respiration refers to the metabolic process in which living organisms (in this case the electroactive biofilm) utilize their own cellular mass as a source of energy instead of relying on new organic matter obtained from their environment, for example, in the absence of an external substrate.

When it comes to benchmarking our MES performance with other electrochemical approaches studied in the literature for 2,4‐D dechlorination, it is not so straightforward as most studies do not report electrode potentials or cell voltage. Furthermore, it should be noted that variables such as reactor design, catalyst loading, catalyst deposition techniques, and actual electrode surface area (rather than projected surface area) are highly sensitive parameters, which can significantly impact the comparison between different technologies. In cases where the cell voltage was not explicitly reported by the authors, it was estimated using polarization curves from low‐temperature electrolysers (Amores et al., 2017; Mandin et al., 2010), in order to calculate energy consumptions.

Table 1 presents the values of the pseudo‐first‐order kinetic constants (*k*_*obs*_, obtained by fitting the experimental data to Equation (8)), energy consumption, and average current efficiency attained in the different works. Similar current efficiency values have been reported in the literature for pure electrochemical reactors dealing with 2,4‐D dechlorination (He et al., 2016; Sun et al., 2015), operating at similar current densities, projected surface area and catholyte volume to the ones used in this work under MEC mode, see Table 1. As already discussed, average current efficiencies were higher under MFC operation since HER was less favoured.

To assess and compare the performance when it comes to 2,4‐D removal/dechlorination rates, pseudo‐first‐order kinetics were proposed and fitted to the experimental data of the works subjected to benchmark. Our MES operating under MEC mode showed a comparable pseudo first order kinetic constant value ($k_{\mathrm{obs}}=4.93\times 10^{-4}\;\min^{-1}$) to the one reported by He et al. (2016) by using a Pd/Ni foam cathode, in which the electrode was prepared through Pd electroless deposition. The same authors improved the performance of the cathode for 2,4‐D dechlorination by using the pulse electrodeposition technique (same Pd loading). The dechlorination rates were enhanced in this latter case, as also shows its *k*_*obs*_, with a value more than two‐fold larger.

The aim of our work was not to optimize the Pd deposition technique, metal substrate or cathode geometry, which could lead to a better dechlorination performance, but to verify the proof of concept of the bio‐anode driven electrodechlorination reaction. Furthermore, it is worth noting that while the projected cathode surface area in this study is comparable to the one employed by He et al. (2016) and Sun et al. (2015), the actual electroactive surface area of the Pd/Ni meshes used in their research is likely larger due to the presence of porosities in the mesh structure. This larger electroactive surface area could account for the higher 2,4‐D removal rate observed in their studies compared to our own results.

Despite the fact that our cathode could be optimized to offer a better dechlorination performance, our MES approach proves to be an interesting option from the point of view of energy consumption, as the biocatalysis allows to operate under a more negative anode potential, favouring the energetic balance. Running the system under MFC mode might have the drawback of being limited to low removal rates, and therefore, longer treatment times; however, energy is produced along the dechlorination process (−0.0120 kWh mol^−1^, the negative symbol indicates that energy is produced). Thus, in order to attain removal rates comparable to conventional (abiotic) electrolysis alternatives, a power source is required, operating the MES a MEC. The energy input under MEC operation was 1.03 kWh mol^−1^, contending the works of He et al. (2016) and Sun et al. (2015). Another critical advantage of our proposal is that the CAPEX (capital expenditure) would be significantly lower, as the anode material used in the MES systems is carbon‐based (i.e., carbon felt). Carbon‐based materials have far lower associated costs compared to the conventional electrochemical processes here evaluated, which use Platinum group metals‐based materials (e.g., Pt, IrO_2_, RuO_2_) for the oxygen evolution reaction (OER) catalysis (otherwise, a higher energy input would be required to overcome the activation overpotential of the OER). Pt prices currently oscillate between 950 and 1000 $/troy oz (30,543–32,150 $/kg) (Johnson Matthey, 2023). Current industrial practices do not use Pt for OER, but generally Nickel, Cobalt or Iron based materials in alkaline electrolysers (Marini et al., 2012; Zeng & Zhang, 2010), or Iridium or Ruthenium based materials in PEM electrolysers (Carmo et al., 2013), which have similar economic implications (prize of Ir and Ru are around 4600 $/troy oz and 465 $/troy oz, respectively) (Johnson Matthey, 2023). Another handicap of noble metal‐based anodes (i.e., platinum group metals, PGMs) is their stability in long‐term operation, as catalyst deactivation and/or dissolution along OER will occur (Schalenbach et al., 2018). For this CAPEX comparison, we assume the cathodic process remains the same in both MES and conventional abiotic electrochemical alternatives that employ Pd‐modified cathodes (the price of Pd is around 1350–1450 $/troy oz). Therefore, the advantage of using this MES configuration (in terms of CAPEX) primarily lies in the inexpensive anode material to support the bioelectrocatalysis.

The biocatalysis favours the oxidation of organics at the anode. In other words, the electroactive biofilm allows to achieve a starting anode potential for the oxidation of the fuel (acetate in this case) closer to the standard potential, decreasing the activation energy or overpotential for such reactions. However, the complex enzymatic processes within the electroactive bacteria do not allow to operate at high current densities, see for example Figure S2b. The (bio)anode performance reaches limiting current conditions (around 0.7 mA cm^−2^) at more positive potentials than −0.2 V_Ag/AgCl_, potential and current density values that were reached by operating under MEC mode at a cell voltage of 0.6 V (Figures 4A and 5B). A higher cell voltage (or a more positive anode polarization) would not have led to significantly higher current densities by the MES, nor faster removal rates. In addition, very positive anode potentials (or large current densities) might lead to the biofilm disruption because of oxidants and/or gasses generation on the electrode (such as O_2_ from water splitting or Cl_2_ from Cl^─^ oxidation, which disproportionates in water and results in hypochlorite formation). Larger current densities can result in local acidic *pH*, and in addition, it can lead to an increase in extracellular substances on the bacterial surface, alter the cell surface charge, reduce the cell growth, inactivate the bacteria, or even lead to cell death (Hoseinzadeh et al., 2020).

Several steps could be taken to improve the performance of the MES. Our cathode material and Pd deposition technique (see Section 2.1) offered an acceptable overall performance and allowed to verify this proof of concept. Nevertheless, better Pd‐based cathode materials (typically using Ni as a substrate), and Pd deposition techniques have been reported in the literature. The use of these cathodes in the MES would enhance their selectivity towards the electro dechlorination reactions, favouring the removal rates.

On the other hand, when working under MEC mode, the bioanode is what limits the system, as its limiting current density value is encountered soon. Therefore, the ratio of anode to cathode projected surface area should be significantly larger. Different configurations could be proposed, that is, using more than one (bio)anode per cell, placing one anode at each side of the planar cathode; or using a tubular reactor configuration, in which the Pd‐based cathode could be a bar or a rod and a cylindrical carbon‐based (bio)anode would be surrounding it, both electrodes being separated by a membrane. In addition, a zero‐gap configuration could also minimize the energy losses of the system.

**FIGURE 7:**
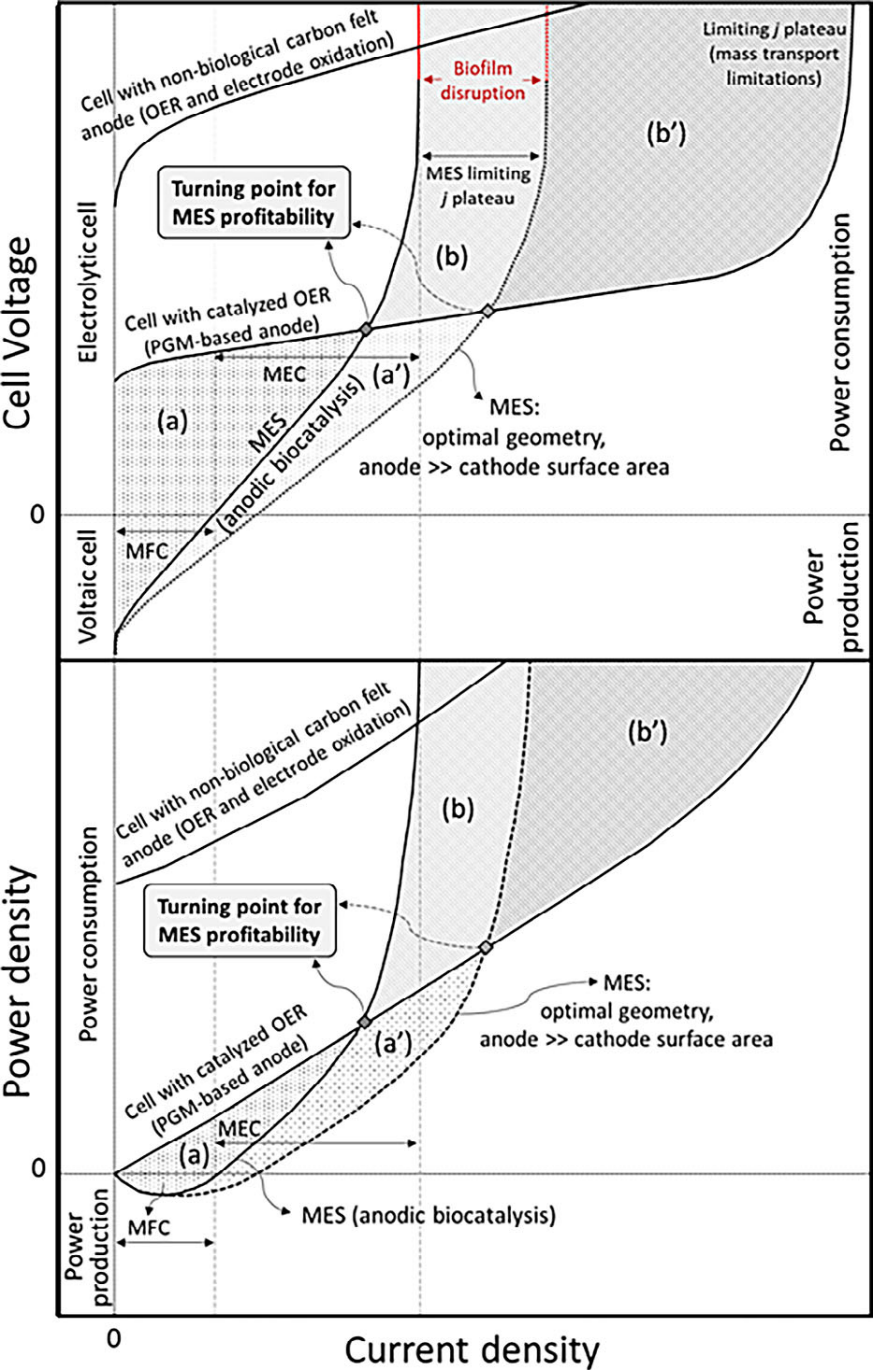
Conceptual performance and profitability analysis of the MES and abiotic electrochemical technologies. Polarization (up) and power (down) curves of the MES and abiotic systems. Different profitability (shaded) regions are depicted: (A) profitability of MES over abiotic systems, (B) profitability of abiotic electrochemical reactor (catalysed OER) over MES, where typical materials for OER catalysis are Pt, IrO_2_, or RuO_2_ (platinum group metals, PGMs).

Compiling the results presented in this paper and analysing previous relevant literature, a landmark turning point can be conceptualized. Figure 7 depicts such a diagram, which describes the regions/operating conditions of profitability of MES versus conventional (abiotic) electrochemical technologies. The intercepts of *Cell voltage* versus *Current density* and *Power density* versus *Current density* curves when comparing an MES case with its abiotic counterpart (carbon felt as anode material or classical PGM‐catalysed OER, using anode materials such as Pt, IrO_2_, or RuO_2_) reveal two essential regions that typify the profitability of one case or another. When the system performs within region (a), the MES technology would be more profitable in terms of OPEX compared to the other cases, as the MES power consumption is lower (with even power production under the MFC operation or voltaic cell region, as displayed in Figure 7). Conversely, when the system performs within region (b), classical electrolysis technology (catalysed OER) would be more advantageous.

The profitability region for the MES can be expanded by operating with a larger ratio of anode to cathode projected surface area or improving the reactor design, obtaining the new regions (a’) and (b’) for MES or classical electrolysis technology, respectively.

Even though this conceptual analysis serves to discuss the profitability region of our microbial electrochemical system, it is extensive to any electrochemical process in which one or both half‐reactions are replaced by bioelectrocatalysis. This comparative diagram intends to shed light on a better understanding of bioelectrochemical technology and to help in the decision‐making to reach good strategies for real potential applications.

### Toxicity and biodegradability

Table 2 reports on the 2,4‐D polluted wastewater biodegradability (quotient between the biological oxygen demand, *BOD*, and the chemical oxygen demand, *COD*) and toxicity (expressed as Half maximal effective concentration, *EC*_*50*_), before and after the bioelectrochemical treatment.

**TABLE 2:** Biodegradability and toxicity of the 2,4‐D synthetic polluted wastewater before and after the treatment through MES technology.

	*BOD*_*f*_ (mg L^−1^)	*COD* (mg L^−1^)	*BOD*_*f*_ */COD*	*EC*_*50*_ (mmol L^−1^)
Before	−5.80	372	−0.016	0.75
After MFC	55	381	0.135	0.93
After MEC	182	395	0.453	1.37

As can be seen, the biodegradability of the original synthetic 2,4‐D polluted wastewater was drastically enhanced. As previously described, the dechlorinated product is less biorefractory. As the dechlorination process was promoted under MEC mode, the best results were attained under this configuration, achieving a $BOD_{f}=182\;\mathrm{mg}\;L^{-1}$ and $BOD_{f}/\mathrm{COD}=0.453$ (the closer to 1 this ratio is, the more biodegradable the sample is). For a typical biodegradable sample, such as municipal wastewater, the $BOD_{f}/\mathrm{COD}$ ratio ranges from 0.4 to 0.6 ($\mathrm{BO}D_{f}$ typically 40–60% of the COD value) (Davis & Cornwell, 2017). The negative *BOD* values of the initial 2,4‐D polluted wastewater give not only an idea of its non‐biodegradability, but also its toxicity. Negative *BOD* values entail endogenous respiration inhibition. This evidences that the MES technology developed in this work can be considered a relevant remediation alternative.

In addition, specific tests were performed to determine the toxicity of the samples by measuring variations in light intensity using inoculated luminescent bacteria, *Aliivibrio fischeri*. The toxicity was evaluated as *EC*_*50*_ (effective concentration that produces 50% of inhibition), in molar concentration. Values of *EC*_*50*_ at 5 min and 15 min were similar, hence only *EC*_*50*_ at 5 min is reported. Higher values of *EC*_*50*_ were attained after the bioelectrochemical treatment, two‐fold higher in the case of the MEC treatment compared to the former stream, evidencing the toxicity reduction after the bio‐assisted dechlorination treatment.

The now more biodegradable effluent would be suitable to undergo conventional aerobic bioprocess treatment to achieve complete mineralization and removal of the organic content (*COD* reduction). Prior to the disposal of such treated streams to the surface waters, they must comply with the current legislation. The European Directive 2008/105/EC on environmental quality standards in the field of water policy discloses a list of priority substances to be monitored in surface waters, including some non‐cyclic, cyclic, and aromatic organochlorines. The limit values for some of these substances are also provided. Table 3 summarizes some relevant cases concerning organochlorines. In agreement with other reported studies (Igbinosa et al., 2013), the higher the content of chlorine atoms in the molecule, the more toxic and biorefractory it is, and therefore, the most restrictive concentration limits in waters shall be set by legislation. Benzene‐kind organochlorines seem to be in the same way less environmentally friendly compared to phenol‐kind ones. Neither the referred directive nor other relevant legislation provide disposal values for 2,4‐D herbicide. As a reference, disposal values could be taken from pentachlorophenol (0.4 μg L^−1^ in inland and other surface waters).

**TABLE 3:** Disposal limit values for some representative organochlorinated compounds (from the European Directive 2008/105/EC on environmental quality standards).

		Annual average value/μg L^−1^		Maximum allowable concentration/μg L^−1^	
Substance	CAS number	Inland surface water	Other surface water	Inland surface water	Other surface water
Hexachloro‐cyclohexane (i.e., Lindane)	608‐73‐1	0.02	0.002	0.04	0.02
Hexachloro‐butadiene	87‐68‐3	‐	‐	0.6	0.6
1,2‐Dichloroethane	107‐06‐2	10	10	Not applicable	Not applicable
Trichloro‐ethylene	79‐01‐6	10	10	Not applicable	Not applicable
Pentachloro‐phenol	87‐86‐5	0.4	0.4	1	1
Pentachloro‐benzene	608‐93‐5	0.007	0.0007	Not applicable	Not applicable
Trichloro‐benzenes	12002‐48‐1	0.4	0.4	Not applicable	Not applicable

## CONCLUSIONS

This work demonstrates the feasibility of cathodic electrodechlorination of 2,4‐D coupled with a bioanode, operating spontaneously under microbial fuel cell mode. However, the dechlorination rates were further enhanced by supplying external voltage (0.6 V), in which 79% dechlorination after 72 h of operation was attained. It is to be noted that no dechlorination took place in the abiotic blanks, revealing the effectiveness of the anodic biocatalysis for driving ECH mechanisms.

The biodegradability (expressed as the ratio *BOD/COD*) of the synthetic 2,4‐D polluted wastewater was increased from negative values (corresponding to toxic effluents) up to 0.453 in the MEC system, evidencing that the MES technology developed in this work can be considered as a relevant remediation alternative.

Our MES approach proves to be a favourable option from the point of view of energy consumption. Running the system under MFC mode allowed to co‐generate energy along the dechlorination process (−0.0120 kWh mol^−1^), even though low removal rates were attained. The energy input under MEC operation was 1.03 kWh mol^−1^—a competitive value compared to previous works reported in the literature for (non‐biological) electrochemical reactors for 2,4‐D electrodechlorination. In addition, the CAPEX (capital expenditure) would be significantly lower in the MES, as the anode material used is carbon‐based (i.e., carbon felt) in contrast to the PGM‐based anodes for oxygen evolution reaction catalysis. In addition, a conceptual landmark turning point for MES versus abiotic electrochemical technologies profitability was discussed.

Several steps could be taken to improve the performance of the MES. Our cathode material (Pd/C on a carbon cloth electrode prepared by drop‐casting) offered an acceptable overall performance and allowed verifying the proof of concept of coupling the bio‐electroactive anode with the cathodic electrodechlorination. Still, better‐performing cathode materials and catalyst deposition techniques have already been reported. The use of these cathodes in the MES would enhance their selectivity towards electrodechlorination reactions, favouring the removal rates. In addition, higher ratios of anode to cathode projected surface area would lead to higher achievable removal rates.

## AUTHOR CONTRIBUTIONS

**Luis F. Leon‐Fernandez:** Conceptualization (equal); data curation (equal); formal analysis (equal); investigation (equal); methodology (equal); writing – original draft (equal); writing – review and editing (equal). **Xochitl Dominguez‐Benetton:** Formal analysis (equal); writing – review and editing (equal). **José Villaseñor Camacho:** Conceptualization (equal); project administration (equal); supervision (equal). **Francisco Jesús Fernandez‐Morales:** Conceptualization (equal); project administration (equal); supervision (equal); writing – review and editing (equal).

## CONFLICT OF INTEREST STATEMENT

The authors declare that they have no known competing financial interests or personal relationships that could have appeared to influence the work reported in this paper.

## Supporting information


**Data S1:** Supporting information.

## Data Availability

The data used in this study are available upon request to the corresponding author.
