# Supplementary material for: Coupling the electrocatalytic dechlorination of 2,4‐D with electroactive microbial anodes
Source: Environ Microbiol Rep. 2023 Jul 21;15(6):512–29. doi: 10.1111/1758-2229.13187 (PMC10667633; doi:10.1111/1758-2229.13187)
Supplement: Supplementary file 1 — Data S1: Supporting information. [file EMI4-15-512-s001.docx]

**Coupling the Electrocatalytic Dechlorination of 2,4-D with Electroactive Microbial Anodes – Supplementary information**

*Luis Fernando Leon-Fernandez^1,2^*, Xochitl Dominguez-Benetton^2^, José Villaseñor^1^, F.J. Fernandez-Morales^1^*

^1^ Chemical Engineering Department, ITQUIMA, University of Castilla-La Mancha, Avenida Camilo José Cela S/N. 13071 Ciudad Real, Spain.

^2^ Separation and Conversion Technologies, Flemish Institute for Technological Research (VITO), Boeretang 200, 2400, Mol, Belgium.

* Corresponding author: Luis Fernando León Fernández

Separation and Conversion Technologies, Flemish Institute for Technological Research (VITO), Boeretang 200, 2400, Mol, Belgium.

Tel: +32 14 33 55 92

E-mail: luis.leon@vito.be

Orcid iD: 0000-0002-9117-1600

**Figure S1.** X-ray diffraction analysis of the carbon cloth electrode with 0.5 mg cm^-2^ Pd loading.

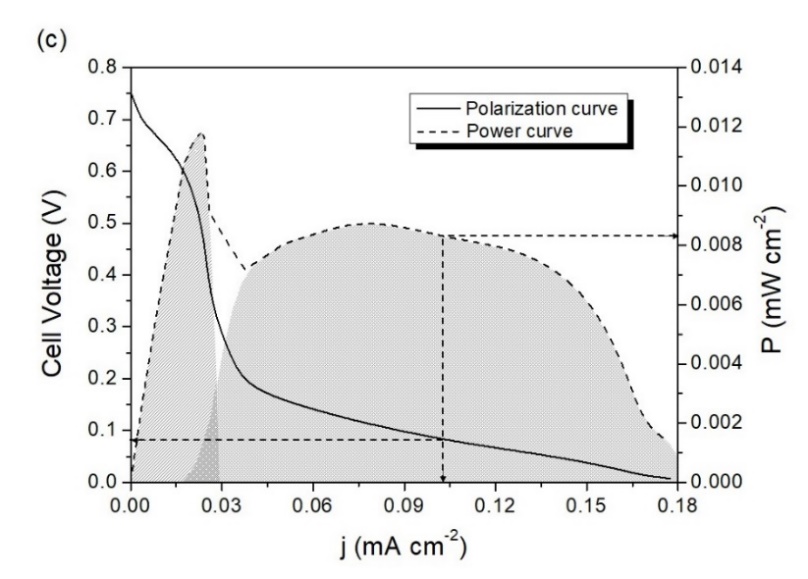


**Figure S2.** (a) LSV for 2.5 x 2.5 cm^2^ carbon cloth electrode with 0.5 mg cm^-2^ Pd loading, 350 rpm magnetic stirring. (b) LSV for 2.5 x 2.5 x 0.8 cm^3^ carbon felt with electroactive biofilm, 60 rpm magnetic stirring. (c) Polarization and Power curves performed to the MFCs. (d) Monitoring of anode and cathode potential throughout the performance of the polarization curve test.

**Table S1.** (Self-)diffusion coefficient of relevant (chlorinated) aromatic compounds

| Compound | Diffusion coefficient / $m s^{-1}$ | Reference |
| --- | --- | --- |
| 2,4-D | $0.58\cdot{10}^{-9}$($T=23 ℃$) | (Scott & Phillips, 1973) |
| 2,4-DCP | $0.78\cdot{10}^{-9}$($T=25 ℃$) | (Martins et al., 2015) |
| Phenol | $1.03\cdot{10}^{-9}$($T=25 ℃$) | (Winkelmann, 2018c) |
| 2-chlorophenol | $0.93\cdot{10}^{-9}$($T=25 ℃$) | (Winkelmann, 2018a) |
| Benzene | $1.02\cdot{10}^{-9}$($T=20 ℃$) | (Lide, 2009) |
|  | $1.1\cdot{10}^{-9}$($T=25 ℃$) | (Winkelmann, 2018b) |
| Benzoic acid | $0.91\cdot{10}^{-9}$($T=20 ℃$) | (Delgado, 2007) |
|  | $1.01\cdot{10}^{-9}$($T=25 ℃$) |  |
| Cyclohexane | $0.84\cdot{10}^{-9}$($T=20 ℃$) | (Lide, 2009) |

**References supplementary material**

Delgado, J. 2007. Molecular Diffusion Coefficients of Organic Compounds in Water at Different Temperatures. *Journal of Phase Equilibria and Diffusion*, **28**, 427-432.

Lide, D.R. 2009. *CRC Handbook of Chemistry and Physics, 90th Edition*. Taylor & Francis.

Martins, L.F.G., Parreira, M.C.B., Ramalho, J.P.P., Morgado, P., Filipe, E.J.M. 2015. Prediction of diffusion coefficients of chlorophenols in water by computer simulation. *Fluid Phase Equilibria*, **396**, 9-19.

Scott, H.D., Phillips, R.E. 1973. Self-Diffusion Coefficients of Selected Herbicides in Water and Estimates of Their Transmission Factors in Soil. *Soil Science Society of America Journal*, **37**(6), 965-967.

Winkelmann, J. 2018a. Diffusion coefficient of 2-chloro-phenol in water, pp. 740-740.

Winkelmann, J. 2018b. Diffusion coefficient of benzene in water at infinite dilution, pp. 2373-2374.

Winkelmann, J. 2018c. Diffusion coefficient of phenol in water, pp. 841-843.
